# 4-[4-(4-Nitro­phenyl­diazen­yl)phen­yl]hexa­nenitrile

**DOI:** 10.1107/S1600536809000853

**Published:** 2009-01-17

**Authors:** Ran-zhe Lu, Lu-na Han, Min Zhang, Bin Wang, Hai-bo Wang

**Affiliations:** a College of Science, Nanjing University of Technology, Xinmofan Road No.5 Nanjing, Nanjing 210009, People’s Republic of China

## Abstract

In the mol­ecule of the title compound, C_18_H_18_N_4_O_2_, the aromatic rings are oriented at a dihedral angle of 3.72 (3)°. In the crystal structure, inter­molecular C—H⋯O hydrogen bonds link the mol­ecules into centrosymmetric dimers. There are also C—H⋯π inter­actions.

## Related literature

For general background, see: Bach *et al.* (1996 ▶); Clark & Hester (1991 ▶); Taniike *et al.* (1996 ▶). For a related structure, see: Zhao *et al.* (2002 ▶). For bond-length data, see: Allen *et al.* (1987 ▶).
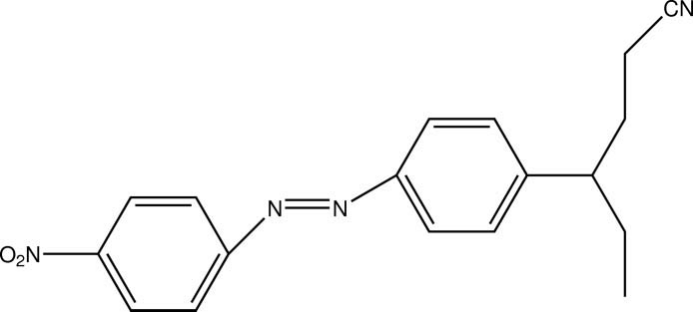


## Experimental

### 

#### Crystal data

C_18_H_18_N_4_O_2_
*M* *_r_* = 322.36
Monoclinic, 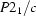

*a* = 20.113 (4) Å
*b* = 10.590 (2) Å
*c* = 7.6820 (15) Å
β = 94.78 (3)°
*V* = 1630.5 (6) Å^3^
*Z* = 4
Mo *K*α radiation
μ = 0.09 mm^−1^
*T* = 293 (2) K
0.30 × 0.20 × 0.10 mm

#### Data collection

Enraf–Nonius CAD-4 diffractometer
Absorption correction: ψ scan (North *et al.*, 1968 ▶) *T*_min_ = 0.964, *T*_max_ = 0.991
3037 measured reflections
2951 independent reflections
1336 reflections with *I* > 2σ(*I*)
*R*_int_ = 0.061
3 standard reflections every 200 reflections intensity decay: 1%

#### Refinement

*R*[*F* ^2^ > 2σ(*F* ^2^)] = 0.072
*wR*(*F* ^2^) = 0.185
*S* = 0.98
2951 reflections
199 parameters
62 restraints
H-atom parameters constrained
Δρ_max_ = 0.32 e Å^−3^
Δρ_min_ = −0.37 e Å^−3^

### 

Data collection: *CAD-4 Software* (Enraf–Nonius, 1989 ▶); cell refinement: *CAD-4 Software*; data reduction: *XCAD4* (Harms & Wocadlo, 1995 ▶); program(s) used to solve structure: *SHELXS97* (Sheldrick, 2008 ▶); program(s) used to refine structure: *SHELXL97* (Sheldrick, 2008 ▶); molecular graphics: *ORTEP-3 for Windows* (Farrugia, 1997 ▶) and *PLATON* (Spek, 2003 ▶); software used to prepare material for publication: *SHELXTL* (Sheldrick, 2008 ▶).

## Supplementary Material

Crystal structure: contains datablocks D, I. DOI: 10.1107/S1600536809000853/hk2611sup1.cif

Structure factors: contains datablocks I. DOI: 10.1107/S1600536809000853/hk2611Isup2.hkl

Additional supplementary materials:  crystallographic information; 3D view; checkCIF report

## Figures and Tables

**Table 1:** Hydrogen-bond geometry (Å, °)

*D*—H⋯*A*	*D*—H	H⋯*A*	*D*⋯*A*	*D*—H⋯*A*
C2—H2*C*⋯O1^i^	0.97	2.46	3.343 (6)	150
C9—H9*A*⋯*Cg*1^ii^	0.93	2.92	3.713 (6)	144
C15—H15*A*⋯*Cg*2^ii^	0.93	2.90	3.690 (6)	143

Symmetry codes: (i) 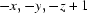; (ii) 
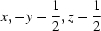
. *Cg*1 and *Cg*2 are the centroids of the C7–C13 and C13–C18 rings, respectively.

**Fig. 1.**
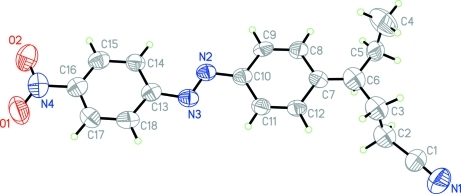
The molecular structure of the title molecule, with the atom-numbering scheme. Displacement ellipsoids are drawn at the 30% probability level.

**Fig. 2.**
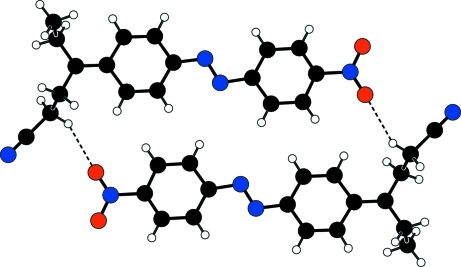
A partial packing diagram of the title compound. Hydrogen bonds are shown as dashed lines.

## supplementary crystallographic information

### Comment

The photophysical properties of azo compounds are of large interest in the development of nonlinear optical and optical data storage materials (Bach *et al.*,  1996; Taniike *et al.*,  1996; Clark & Hester, 1991). As part of our studies in this area, we report herein the synthesis and crystal structure of the title compound.

In the molecule of the title compound (Fig 1), the bond lengths (Allen *et al.*,  1987) and angles are within normal ranges. Rings A (C7-C12) and B (C13-C18) are, of course, planar, and they are oriented at a dihedral angle of 3.72 (3)°.

In the crystal structure, intermolecular C-H···O hydrogen bonds (Table 1) link the molecules into centrosymmetric dimers (Fig. 2), in which they may be effective in the stabilization of the structure. There also exist C–H···π interactions (Table 1).

### Experimental

The title compound has been synthesized according to a literature method (Zhao *et al.*,  2002). Crystals suitable for X-ray analysis were obtained by slow evaporation of an ethanol solution.

### Refinement

H atoms were positioned geometrically, with C-H = 0.93, 0.97 and 0.96 Å for aromatic, methylene and methyl H, respectively, and constrained to ride on their parent atoms, with U_iso_(H) = xU_eq_(C), where x = 1.5 for methyl H and x = 1.2 for all other H atoms.

### Crystal data

**Table:**

C_18_H_18_N_4_O_2_	*F*(000) = 680
*M**_r_* = 322.36	*D*_x_ = 1.313 Mg m^−^^3^
Monoclinic, *P*2_1_/*c*	Mo *K*α radiation, λ = 0.71073 Å
Hall symbol: -P 2ybc	Cell parameters from 25 reflections
*a* = 20.113 (4) Å	θ = 9–13°
*b* = 10.590 (2) Å	µ = 0.09 mm^−^^1^
*c* = 7.6820 (15) Å	*T* = 293 K
β = 94.78 (3)°	Block, red
*V* = 1630.5 (6) Å^3^	0.30 × 0.20 × 0.10 mm
*Z* = 4	

### Data collection

**Table:**

Enraf–Nonius CAD-4 diffractometer	1336 reflections with *I* > 2σ(*I*)
Radiation source: fine-focus sealed tube	*R*_int_ = 0.061
graphite	θ_max_ = 25.3°, θ_min_ = 1.0°
ω/2θ scans	*h* = 0→24
Absorption correction: ψ scan (North *et al.*, 1968)	*k* = 0→12
*T*_min_ = 0.964, *T*_max_ = 0.991	*l* = −9→9
3037 measured reflections	3 standard reflections every 200 reflections
2951 independent reflections	intensity decay: 1%

### Refinement

**Table:**

Refinement on *F*^2^	Primary atom site location: structure-invariant direct methods
Least-squares matrix: full	Secondary atom site location: difference Fourier map
*R*[*F*^2^ > 2σ(*F*^2^)] = 0.072	Hydrogen site location: inferred from neighbouring sites
*wR*(*F*^2^) = 0.185	H-atom parameters constrained
*S* = 0.98	*w* = 1/[σ^2^(*F*_o_^2^) + (0.060*P*)^2^ + 1.*P*] where *P* = (*F*_o_^2^ + 2*F*_c_^2^)/3
2951 reflections	(Δ/σ)_max_ < 0.001
199 parameters	Δρ_max_ = 0.32 e Å^−^^3^
62 restraints	Δρ_min_ = −0.37 e Å^−^^3^

### Special details

**Table:**

Geometry. All esds (except the esd in the dihedral angle between two l.s. planes) are estimated using the full covariance matrix. The cell esds are taken into account individually in the estimation of esds in distances, angles and torsion angles; correlations between esds in cell parameters are only used when they are defined by crystal symmetry. An approximate (isotropic) treatment of cell esds is used for estimating esds involving l.s. planes.
Refinement. Refinement of F^2^ against ALL reflections. The weighted R-factor wR and goodness of fit S are based on F^2^, conventional R-factors R are based on F, with F set to zero for negative F^2^. The threshold expression of F^2^ > 2sigma(F^2^) is used only for calculating R-factors(gt) etc. and is not relevant to the choice of reflections for refinement. R-factors based on F^2^ are statistically about twice as large as those based on F, and R- factors based on ALL data will be even larger.

### Fractional atomic coordinates and isotropic or equivalent isotropic displacement parameters (Å^2^)

**Table:**

	*x*	*y*	*z*	*U*_iso_*/*U*_eq_	
N1	0.4653 (3)	−0.1899 (5)	0.4255 (7)	0.1057 (17)	
C1	0.4327 (3)	−0.1281 (7)	0.4966 (8)	0.107 (2)	
O1	−0.27421 (18)	0.0779 (4)	0.7659 (5)	0.0975 (13)	
O2	−0.25317 (17)	0.2141 (3)	0.9614 (5)	0.0828 (11)	
N2	0.07709 (17)	0.1393 (3)	0.8661 (4)	0.0479 (7)	
N3	0.03734 (18)	0.0691 (3)	0.7775 (4)	0.0506 (9)	
N4	−0.2356 (2)	0.1355 (4)	0.8566 (6)	0.0658 (11)	
C2	0.3903 (2)	−0.0199 (5)	0.5602 (7)	0.0829 (10)	
H2B	0.4142	0.0597	0.5607	0.099*	
H2C	0.3493	−0.0113	0.4853	0.099*	
C3	0.3747 (2)	−0.0512 (5)	0.7350 (7)	0.0829 (10)	
H3B	0.4143	−0.0822	0.8022	0.099*	
H3C	0.3411	−0.1171	0.7309	0.099*	
C4	0.4104 (3)	0.0772 (6)	1.1039 (8)	0.105 (2)	
H4A	0.4447	0.1228	1.1719	0.157*	
H4B	0.4239	−0.0091	1.0920	0.157*	
H4C	0.3698	0.0801	1.1613	0.157*	
C5	0.3989 (2)	0.1368 (5)	0.9246 (7)	0.0808 (17)	
H5A	0.3851	0.2240	0.9360	0.097*	
H5B	0.4400	0.1358	0.8671	0.097*	
C6	0.3485 (2)	0.0682 (5)	0.8228 (7)	0.0829 (10)	
H6A	0.3520	0.1208	0.7190	0.099*	
C7	0.2832 (2)	0.0858 (4)	0.8230 (6)	0.0583 (12)	
C8	0.2566 (2)	0.1767 (4)	0.9332 (5)	0.0519 (11)	
H8A	0.2857	0.2281	1.0017	0.062*	
C9	0.1910 (2)	0.1910 (4)	0.9421 (5)	0.0504 (11)	
H9A	0.1763	0.2521	1.0169	0.060*	
C10	0.1431 (2)	0.1179 (4)	0.8435 (5)	0.0479 (7)	
C11	0.1685 (2)	0.0266 (4)	0.7336 (5)	0.0498 (11)	
H11A	0.1390	−0.0263	0.6687	0.060*	
C12	0.2337 (2)	0.0142 (4)	0.7206 (5)	0.0506 (11)	
H12A	0.2479	−0.0439	0.6408	0.061*	
C13	−0.0292 (2)	0.0918 (4)	0.8074 (5)	0.0470 (10)	
C14	−0.0524 (2)	0.1820 (4)	0.9173 (5)	0.0511 (11)	
H14A	−0.0223	0.2352	0.9798	0.061*	
C15	−0.1198 (2)	0.1937 (4)	0.9352 (6)	0.0588 (12)	
H15A	−0.1348	0.2540	1.0108	0.071*	
C16	−0.1639 (2)	0.1184 (4)	0.8440 (5)	0.0521 (11)	
C17	−0.1442 (2)	0.0243 (4)	0.7373 (6)	0.0583 (12)	
H17A	−0.1753	−0.0289	0.6783	0.070*	
C18	−0.0770 (2)	0.0112 (4)	0.7203 (5)	0.0618 (12)	
H18A	−0.0627	−0.0528	0.6493	0.074*	

### Atomic displacement parameters (Å^2^)

**Table:**

	*U*^11^	*U*^22^	*U*^33^	*U*^12^	*U*^13^	*U*^23^
N1	0.100 (4)	0.112 (4)	0.104 (4)	0.046 (3)	0.004 (3)	−0.022 (3)
C1	0.064 (4)	0.138 (6)	0.118 (5)	0.009 (4)	0.005 (3)	−0.064 (5)
O1	0.058 (2)	0.128 (3)	0.105 (3)	−0.018 (2)	−0.005 (2)	0.007 (3)
O2	0.073 (2)	0.077 (3)	0.101 (3)	0.017 (2)	0.022 (2)	0.008 (2)
N2	0.0573 (17)	0.0468 (16)	0.0392 (14)	0.0041 (14)	0.0020 (13)	0.0020 (13)
N3	0.060 (2)	0.049 (2)	0.043 (2)	0.0021 (18)	−0.0003 (17)	0.0016 (17)
N4	0.060 (3)	0.061 (3)	0.076 (3)	0.004 (2)	0.004 (2)	0.023 (2)
C2	0.069 (2)	0.093 (2)	0.086 (2)	−0.0043 (17)	−0.0003 (17)	−0.010 (2)
C3	0.069 (2)	0.093 (2)	0.086 (2)	−0.0043 (17)	−0.0003 (17)	−0.010 (2)
C4	0.086 (4)	0.112 (5)	0.111 (5)	−0.048 (4)	−0.023 (4)	0.008 (4)
C5	0.042 (3)	0.110 (4)	0.091 (4)	−0.014 (3)	0.010 (3)	−0.046 (4)
C6	0.069 (2)	0.093 (2)	0.086 (2)	−0.0043 (17)	−0.0003 (17)	−0.010 (2)
C7	0.051 (3)	0.069 (3)	0.055 (3)	−0.007 (2)	−0.001 (2)	−0.017 (2)
C8	0.055 (3)	0.040 (2)	0.061 (3)	−0.007 (2)	0.006 (2)	−0.019 (2)
C9	0.064 (3)	0.047 (3)	0.041 (2)	−0.001 (2)	0.011 (2)	−0.014 (2)
C10	0.0573 (17)	0.0468 (16)	0.0392 (14)	0.0041 (14)	0.0020 (13)	0.0020 (13)
C11	0.052 (3)	0.052 (3)	0.044 (2)	0.003 (2)	−0.003 (2)	−0.005 (2)
C12	0.056 (3)	0.044 (2)	0.052 (3)	−0.001 (2)	0.000 (2)	−0.016 (2)
C13	0.059 (3)	0.044 (2)	0.038 (2)	0.005 (2)	0.0057 (19)	0.0108 (19)
C14	0.054 (3)	0.051 (3)	0.048 (3)	0.006 (2)	−0.002 (2)	−0.005 (2)
C15	0.070 (3)	0.056 (3)	0.051 (3)	0.012 (2)	0.005 (2)	−0.005 (2)
C16	0.065 (3)	0.048 (3)	0.044 (2)	0.002 (2)	0.007 (2)	0.014 (2)
C17	0.057 (3)	0.058 (3)	0.058 (3)	−0.005 (2)	−0.005 (2)	0.003 (2)
C18	0.075 (3)	0.066 (3)	0.045 (2)	0.001 (3)	0.003 (2)	−0.007 (2)

### Geometric parameters (Å, °)

**Table:**

N1—C1	1.102 (6)	C6—H6A	0.9800
C1—C2	1.533 (7)	C7—C8	1.416 (5)
O1—N4	1.171 (5)	C7—C12	1.434 (5)
O2—N4	1.230 (5)	C8—C9	1.334 (5)
N2—N3	1.251 (4)	C8—H8A	0.9300
N2—C10	1.371 (5)	C9—C10	1.407 (5)
N3—C13	1.398 (5)	C9—H9A	0.9300
N4—C16	1.464 (6)	C10—C11	1.407 (5)
C2—C3	1.443 (6)	C11—C12	1.330 (5)
C2—H2B	0.9700	C11—H11A	0.9300
C2—H2C	0.9700	C12—H12A	0.9300
C3—C6	1.546 (7)	C13—C14	1.381 (5)
C3—H3B	0.9700	C13—C18	1.412 (6)
C3—H3C	0.9700	C14—C15	1.379 (5)
C4—C5	1.515 (7)	C14—H14A	0.9300
C4—H4A	0.9600	C15—C16	1.346 (6)
C4—H4B	0.9600	C15—H15A	0.9300
C4—H4C	0.9600	C16—C17	1.369 (6)
C5—C6	1.427 (6)	C17—C18	1.376 (6)
C5—H5A	0.9700	C17—H17A	0.9300
C5—H5B	0.9700	C18—H18A	0.9300
C6—C7	1.326 (6)		
			
N1—C1—C2	166.2 (8)	C6—C7—C8	121.3 (4)
N3—N2—C10	114.5 (3)	C6—C7—C12	124.6 (4)
N2—N3—C13	112.7 (3)	C8—C7—C12	114.0 (4)
O1—N4—O2	122.0 (5)	C9—C8—C7	122.1 (4)
O1—N4—C16	120.3 (5)	C9—C8—H8A	118.9
O2—N4—C16	117.7 (5)	C7—C8—H8A	118.9
C3—C2—C1	107.1 (5)	C8—C9—C10	123.1 (4)
C3—C2—H2B	110.0	C8—C9—H9A	118.4
C1—C2—H2B	111.2	C10—C9—H9A	118.4
C3—C2—H2C	109.5	N2—C10—C9	117.9 (4)
C1—C2—H2C	110.4	N2—C10—C11	126.4 (4)
H2B—C2—H2C	108.6	C9—C10—C11	115.7 (4)
C2—C3—C6	109.1 (5)	C12—C11—C10	121.5 (4)
C2—C3—H3B	109.9	C12—C11—H11A	119.2
C6—C3—H3B	109.9	C10—C11—H11A	119.2
C2—C3—H3C	109.9	C11—C12—C7	123.4 (4)
C6—C3—H3C	109.9	C11—C12—H12A	118.3
H3B—C3—H3C	108.3	C7—C12—H12A	118.3
C5—C4—H4A	109.5	C14—C13—N3	126.6 (4)
C5—C4—H4B	109.5	C14—C13—C18	117.2 (4)
H4A—C4—H4B	109.5	N3—C13—C18	116.2 (4)
C5—C4—H4C	109.5	C15—C14—C13	120.5 (4)
H4A—C4—H4C	109.5	C15—C14—H14A	119.7
H4B—C4—H4C	109.5	C13—C14—H14A	119.7
C6—C5—C4	109.5 (5)	C16—C15—C14	120.3 (4)
C6—C5—H5A	109.8	C16—C15—H15A	119.8
C4—C5—H5A	109.8	C14—C15—H15A	119.8
C6—C5—H5B	109.8	C15—C16—C17	122.1 (4)
C4—C5—H5B	109.8	C15—C16—N4	120.1 (4)
H5A—C5—H5B	108.2	C17—C16—N4	117.7 (4)
C7—C6—C5	125.9 (5)	C16—C17—C18	117.8 (4)
C7—C6—C3	119.3 (5)	C16—C17—H17A	121.1
C5—C6—C3	113.8 (4)	C18—C17—H17A	121.1
C7—C6—H6A	93.4	C17—C18—C13	121.9 (4)
C5—C6—H6A	93.4	C17—C18—H18A	119.0
C3—C6—H6A	93.4	C13—C18—H18A	119.0
			
C10—N2—N3—C13	178.6 (3)	C10—C11—C12—C7	3.9 (6)
N1—C1—C2—C3	−168 (3)	C6—C7—C12—C11	174.3 (5)
C1—C2—C3—C6	165.2 (4)	C8—C7—C12—C11	−3.4 (6)
C4—C5—C6—C7	85.4 (7)	N2—N3—C13—C14	1.4 (5)
C4—C5—C6—C3	−82.7 (6)	N2—N3—C13—C18	−176.3 (3)
C2—C3—C6—C7	97.0 (6)	N3—C13—C14—C15	−179.6 (4)
C2—C3—C6—C5	−94.1 (6)	C18—C13—C14—C15	−2.0 (6)
C5—C6—C7—C8	−1.8 (9)	C13—C14—C15—C16	−0.8 (6)
C3—C6—C7—C8	165.7 (4)	C14—C15—C16—C17	3.1 (6)
C5—C6—C7—C12	−179.3 (5)	C14—C15—C16—N4	−176.9 (4)
C3—C6—C7—C12	−11.8 (8)	O1—N4—C16—C15	173.2 (4)
C6—C7—C8—C9	−176.3 (5)	O2—N4—C16—C15	−5.0 (6)
C12—C7—C8—C9	1.4 (6)	O1—N4—C16—C17	−6.8 (6)
C7—C8—C9—C10	−0.1 (7)	O2—N4—C16—C17	175.1 (4)
N3—N2—C10—C9	−178.4 (3)	C15—C16—C17—C18	−2.3 (6)
N3—N2—C10—C11	−0.3 (6)	N4—C16—C17—C18	177.6 (4)
C8—C9—C10—N2	178.7 (4)	C16—C17—C18—C13	−0.7 (6)
C8—C9—C10—C11	0.4 (6)	C14—C13—C18—C17	2.7 (6)
N2—C10—C11—C12	179.6 (4)	N3—C13—C18—C17	−179.4 (4)
C9—C10—C11—C12	−2.2 (6)		

### Hydrogen-bond geometry (Å, °)

**Table:**

*D*—H···*A*	*D*—H	H···*A*	*D*···*A*	*D*—H···*A*
C2—H2C···O1^i^	0.97	2.46	3.343 (6)	150
C9—H9A···Cg1^ii^	0.93	2.92	3.713 (6)	144
C15—H15A···Cg2^ii^	0.93	2.90	3.690 (6)	143

Symmetry codes: (i) −*x*, −*y*, −*z*+1; (ii) *x*, −*y*−1/2, *z*−1/2.
